# Key informants perspectives on creating a high impact research department in family and community medicine: a qualitative project

**DOI:** 10.1186/s12875-024-02288-6

**Published:** 2024-02-06

**Authors:** Allison Gayapersad, Mary Ann O’Brien, Christopher Meaney, Ishan Aditya, Julia Baxter, Peter Selby

**Affiliations:** 1 INTREPID Lab, Centre for Addiction and Mental Health, Toronto, Ontario Canada; 2 Department of Family and Community Medicine, University of Toronto, Toronto, Ontario Canada; 3 Campbell Family Mental Health Research Institute, Centre for Addiction and Mental Health, Toronto, Ontario Canada; 4 Dalla Lana School of Public Health, University of Toronto, Toronto, Ontario Canada

**Keywords:** High impact research, Qualitative, Primary care, Socioecological framework

## Abstract

**Background:**

Primary care is integral to the health system and population health. Primary care research is still in development and most academic departments lack effective research investments. High impact primary care research programs are needed to advance the field to ensure a robust primary care system for the future. The project objective was to understand key informants’ views of structures, functions, and processes required to create a high impact research program in an academic primary care department.

**Methods:**

A descriptive qualitative project with key informants from research programs in primary care. Participants included international research leaders in primary care (*n* = 10), department of family and community researchers (*n* = 37) and staff (*n* = 9) in an academic primary care department, other university leaders (*n* = 3) and members of the departmental executive leadership team (1 department; 25 members). Semi-structured interviews (*n* = 27), and focus groups (*n* = 6) were audio recorded, transcribed, and analyzed using thematic analysis. We used a socioecological framework which described micro, meso, macro levels of influence.

**Results:**

At the micro level despite barriers with respect to funding, protected time and lack of formal mentorship, personal motivation was a key factor. At the meso level, the organizational structure that promoted collaboration and a sense of connection emerged as a key factor. Specifically research leaders identified a research faculty development pipeline based on equity, diversity, inclusion, indigeneity, and accessibility principles with thematic areas of focus as key enablers. Lastly, at the macro level, an overarching culture and policies that promoted funding and primary care research was associated with high impact programs.

**Conclusion:**

The alignment/complementarity of micro, meso, and macro level factors influenced the creation of a high impact research department in primary care. High impact research in primary care is facilitated by the development of researchers through formalized and structured mentorship/sponsorship and a department culture that promote primary care research.

**Supplementary Information:**

The online version contains supplementary material available at 10.1186/s12875-024-02288-6.

## Background

The 1978 Alma-Ata Declaration recognized the importance of primary care as integral to the health system and the overall social and economic development of the community. In addition, the importance of primary care research was emphasized [1]. The broad scope of primary care research has the potential to address the health of individuals and populations through a collaborative approach to basic, clinical, health services, health systems, public health, community health and well being, disease prevention, health promotion, and educational research [2]. Across the lifespan, a patients’ first point of contact and subsequent care within the health care system is with their primary care providers [3]. Thus primary care providers should have a strong role in primary care research given their unique perspectives on individuals, families and communities [2, 4]. High impact primary care research that can be disseminated broadly, impact clinical outcomes, and of relevance to underserved clinical populations is needed [5].

Although research has demonstrated the value of primary care in improving the health of populations and reducing health care costs, much remains unknown [6, 7]. The recent advances in electronic medical records (EMRs), data informed care delivery, and learning health systems in primary care require the development of a research agenda that is inclusive, participatory, and reduces disparities in society [6, 7]. Moreover, a focus on equity, diversity, inclusion, indigeneity, and accessibility (EDIIA) in the workforce and the delivery of care in community settings requires study [6, 8, 9]. Primary care settings are where recent advances in science such as genetics and artificial intelligence need to be co-developed, implemented, and studied. Lastly, emerging issues such as planetary health and primary care provider wellness are also important topics of research. Therefore, the future agenda for primary care is sophisticated and needs an interdisciplinary scientist workforce equipped to address identified gaps at clinical and policy levels to improve outcomes for populations served [6–11]. To that end, university primary care academic departments must support the development of primary care clinician scientist workforce and research production [12–14]. Activities include opportunities for training and collaboration, mentorship, and the research and administrative supports needed to create high impact research that goes beyond traditional outputs of grants held and papers published in peer reviewed journals [15].

As part of a larger environmental scan, we conducted a qualitative project to understand key informants’ views of the challenges and enablers to high impact research in an academic department of primary care. The larger environmental scan included a scoping review of research capacity building in academic primary care research departments [16], and a scientometric analysis of primary care research literature [17].

The qualitative project is the focus of this paper which addressed the following specific question, “What structures, functions, and processes are required to create a high impact research program in primary care?” By having a better understanding of these factors, a more informed approach can be taken to strengthen the research program.

## Methods

### Project design

For this qualitative project, we used a socioecological theoretical framework, based on Bronfenbrenner’s Ecological Systems Theory to examine various levels in the environment that interacted and affected the researcher and influenced the factors required to create a high impact primary care research program [18]. We adopted this socioecological framework because it allowed us to consider factors at three levels, the micro, the meso, and the macro, which interact with, and influence one another [18, 19]. The framework can help research programs target multiple/key levels in their setting to address barriers to the creation of high impact research. Strategies targeting multiple levels may have long-lasting impact and be more effective than strategies targeting one level [20]. The framework guided the development of interview and focus group questions (Supplemental File 1). To avoid imposing a definition of a high impact research department given the lack of an accepted definition [21, 22], we asked participants to share their perspectives and the key metrics of research impact. The qualitative research design is particularly well suited to explore various key informants’ perceptions of the challenges and enablers to creating a high impact research program. The Consolidated Criteria for Reporting Qualitative Research guided the writing of this article [23].

### Context

The Department of Family and Community Medicine (DFCM), University of Toronto, has over 2000 faculty members, most are clinicians caring for a defined patient population; secondarily may be involved in clinical teaching, leadership/management, educational scholarship, quality improvement and other roles; with a few participating in health and biomedical research. Thirty faculty members receive a research stipend from the department [24]. There are about 75 (of the 2000) current researchers with an estimate of 225 who have ever been involved in research [25, 26]. The DFCM reports researchers in 2018 received, as primary- and co-investigators, 223 grants of over $50 million in funding and produced over 500 peer-reviewed publications. In addition, DFCM faculty members participated in 104 grants as co-investigators, where the principal investigators were not members of the DFCM [27]. Many of the DFCM teaching sites are part of the Toronto Academic Health Science Network (TAHSN) [28]. The TAHSN comprises nine teaching hospitals fully affiliated with the University of Toronto, four associate member institutions and extends to another 20 community-affiliated hospitals and healthcare sites in the Greater Toronto Area and beyond. The DFCM research program is characterized as a ‘hub and spoke’ organizational model [29]. The hub includes the biostatistical service, which is available to all faculty. DFCM researchers can access support, including administrative support for grant writing, Curriculum Vitae preparation and survey software support, through the DFCM Office of Educational Scholarship and Quality Improvement as well as through their respective teaching hospital. DFCM researchers also have access to data management and governance through the University of Toronto Practice Based Research Network (UTOPIAN). UTOPIAN is a platform for conducting research and providing collaboration and mentorship opportunities. DFCM researchers can access de-identified patient data from contributing practices and collaborate with primary care clinicians, and practices from all DFCM academic sites to answer important healthcare questions and translate findings into practice [30].

### Participants, recruitment, and data collection

We conducted semi-structured interviews and focus groups with key informants which included international research leaders in primary care (*n* = 10) from four US universities (University of North Carolina, University of California, San Francisco, Harvard Medical School, and University of Missouri-Columbia); three universities in the United Kingdom (University of Oxford, University of Glasgow, and University of St. Andrews); one Australian university (University of Melbourne) and a member of the Department of Health, Australia, the Netherlands Institute for Health Services Research; University of Toronto affiliated and department leaders (*n* = 17), early, mid and senior career research faculty (*n* = 23), and DFCM staff (*n* = 9) and consultation with the DFCM Executive Committee (*n* = 1, 25 members), between December 2021 and April 2022. We used snowball sampling to select research leaders and used purposeful sampling to recruit research faculty and staff from the DFCM, University of Toronto and affiliated hospital and community sites. We also purposefully sampled among department leadership of primary care from community sites. We contacted key informants by email to invite them to participate. Interviews and focus groups were conducted using Zoom (audio chat). PS, a physician and acting Vice Chair of Research in DFCM and AG, a PhD qualitative health researcher, together conducted interviews with national and international research leaders. MAO, an experienced qualitative researcher and AG conducted interviews and focus groups with DFCM researchers and research staff. Interviews and focus groups were audio recorded and transcribed. An informed consent statement was not required to be submitted for approval by the University of Toronto (U of T) Research Ethics Boards (REBs), Health Sciences REB as they designated the project as a quality improvement project.

### Data analysis

**Fig. 1:**
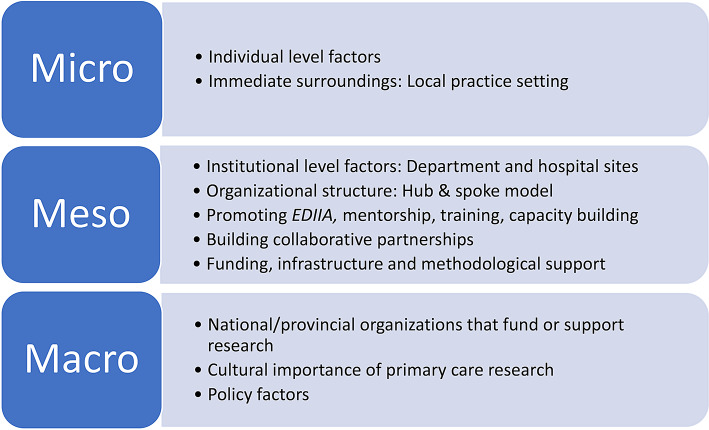
Sociological framework (adapted from Bronfenbrenner, 2005). Bronfenbrenner U. Making human beings human: bioecological perspectives on human development. Thousand Oaks: Sage Publications; 2005. The project used a socio-ecological approach, adapted from Bronfenbrenner (2005), to explore factors that influence research capacity at the micro, meso and macro levels whereby micro refers to the individual; meso refers to Departmental and Hospital sites; and macro refers to national/provincial funding organizations and the overarching culture and policy environment

We used the software, NVivo12, (QSR International) to manage the data analysis process. Researchers (AG/MAO) immersed themselves in the data to determine data driven themes. We used the constant comparative method to analyze the data [31]. Line by line coding of the transcripts led to the creation of initial descriptive codes, which were then clustered into initial categories, followed by refinement of the categories into themes [32]. We reached saturation of the data when no further replication of instances within themes were identified in the transcripts [33]. AG and MAO discussed emergent themes, patterns and connections within and across the transcripts. This process of analysis enabled us to be consistent in our coding, and to refute or clarify interpretations through consensus and reference to the data. Themes were mapped to socio-ecological concepts [18] examining the structures, functions, and processes at the micro, meso, and macro levels (Fig. 1). In addition to this systematic approach to data collection and analysis, we created and maintained an audit trail of coded transcripts and memos and used a range of expertise within the project team to enhance trustworthiness of the project [34].

## Results

Data from twenty-seven key informant interviews and six focus groups (*n* = 32 participants) contained rich insights into the structures, functions and processes that act as barriers or enablers to creating a high impact primary care research department. Using a socio-ecological framework, the findings from this project are presented under three levels of influence: micro, meso and macro. Across the data set, our analysis demonstrated the factors that influenced the production of high impact research at different levels (i.e., micro, meso, macro) which operated within and across these different levels. Due to the focus of the paper, we orientate each level as a main theme with each having cross-cutting sub-themes with illustrative descriptions. Although presented as discrete categories (levels) for clarity it is important to note that these different levels are not mutually exclusive but reinforce and operate on and with each other. Thus, high impact is a product of an ecosystem that nurtures researchers in primary care.

Participants discussed the research enterprise at individual, departmental/institutional, and overarching policy levels, which sometimes overlapped or appeared to be interdependent. At the micro level of influence, we describe DFCM researchers’ experiences of the challenges and enablers to producing high impact research and the creation of a high impact research department. At the meso and macro levels, we described international and University of Toronto research leaders’ perspectives of the key departmental, institutional and policy factors required for the creation of a high impact research department. Before presenting findings for each level, we provide the project researchers’ definition and the key metrics of research impact.

### Defining high impact in primary care research

Researchers in our project believed that it was important to go beyond the traditional measures of research impact and characterized high impact research in primary care as the real world impact this area of research has on the health care system and population health.

I think high impact research in family medicine should be that it’s public facing…. Have a role in changing something…. Real world impact… I think that’s even more important in family medicine research… because we’re the backbone of the health care system. (Early Career Researcher (ECR) Focus Group (FG) 1)

I think impact is actually making a difference…. Uptake of the findings [by] policymakers. A lot of the impact is the change in health care systems. Research that changed a guideline, develop standards for practice that in our area of expertise will change the way family medicine is practiced… (Mid-Career Researcher (MCR) FG).

Researchers agreed that while challenging, metrics of a high impact research department in primary care be established and the research activities measured accordingly.

### Micro level: individual level challenges and enablers

This micro level of influence encompasses the researcher and their local environment, usually the practice setting (the spoke). Themes described are related to individual characteristics such as researchers’ motivation and interpersonal collaborative networks and support system to produce high impact research in their immediate surroundings. Researchers’ motivation was an enabler to the production of high impact research. A do-it-yourself research career pathway and the lack of a supportive research culture were identified as challenges to producing high impact research.

### Motivations to improve patient care through research

Research faculty indicated that their motivation for conducting research was driven by their passion for research, curiosity, and the desire to effect change and improve the lives of patients. Their motivations appeared to be a key enabler for producing high impact research. For example,

And it’s a curiosity thing for me, which is why I love research and what motivates me. It’s wanting to study cause and effect, wanting to make a difference and change the health care system and improve patient care. (MCR FG))

### Do-it-yourself research career pathway

Although considered of significant importance to research success researchers mentioned that they had limited opportunities for mentorship and collaboration. Instead, researchers indicated that their research career path has been one of a ‘do-it-yourself’ endeavor. For some, the path to success appeared to be based on luck. For example,

I think it’s do-it-yourself, but, really, the success is so dependent on mentorship and supports. So, what you see is that if people have that, then they’re succeeding and they’re able to navigate the system. And so, … there’s a high attrition rate, where people start down this path, they see that … there’s this do-it-yourself aspect. But also, if they’re not supported and sheltered … to allow to grow, then it doesn’t end up really bearing fruit over time. (MCR FG)

I’ll share a challenge. I had this idea that if I could find the right research team, I could bring some clinical and content area expertise… we could collaborate… And it was impossible to find. (ECR FG1)

Varying levels of leadership support for primary care research across the academic and community sites reinforce this do-it-yourself endeavor. Research faculty mentioned that the culture at the local setting should be one that valued research, where leaders endorsed the importance of research and were supportive of the research enterprise. Research faculty at community sites found that a supportive research culture was still in the early stages of development compared to academic sites.

When I was a primary care resident, nobody was saying “are you interested in research? We would like to support a research career.” I think people coming in are changing. But then we need to have not only a culture of valuing research. (SCR FG).

…build a culture where people can sort of take some of these projects and things to the next step… to build this culture of curiosity…. There was a chief for eight years… I don’t know how supportive he was of research… And I think some of those… felt a little bit left behind… And we’re really trying to build that culture. (KII 24)

### Meso level: institutional structures, supports and processes

The themes described at the meso and macro levels are related to the wider structural factors that shape the production of high impact research in primary care. The meso level encompasses the organizational characteristics, the functions and processes of the academic department, hospital and community structures, which interact synergistically with the micro level individual characteristics and interpersonal networks and supports, and macro level policy considerations. The first theme identified included the DFCM organizational structure, the hub and spoke model, and its influence on the researcher at the micro level. Other themes identified were related to the key enablers for the creation of a high impact research program: the functions and processes related to creating a pipeline of diverse and skilled researchers, building collaborative networks, resources and infrastructure support, and thematic research focus. A number of these key enablers align with the challenges experienced by DFCM researchers.

### The hub and spoke organizational structure

The international research programs included in this project were diverse and few had a hub and spoke structure (2) similar to the DFCM. Most research programs were not directly affiliated with clinical care. The hub and spoke structure appeared to be a barrier to having a sense of connection to the central department. For example,

We’ve got a wide, vast, decentralized faculty… from the community sites to the fully affiliated sites… Many of us work clinically. I think creating a greater connection to the division [department] is important…. Bringing people in to feel like they belong at the division. (KII 19).

The hub and spoke organizational model may have been a barrier to research collaboration amongst sites.

I think that’s the first thing we have to do, is try to break down those silos. Let’s try to form some collaborations across the [department]. (MCR FG)

Consequently, researchers often identified more with their hospital and its family medicine researcher unit (spoke), creating subcultures of research which impacted identification with the central hub research culture. For example,

The more you have an allegiance or the culture, and you identify more with your hospital, family medicine hospital research unit…. And to me, if it lacks the vision… overall leadership from [central department]… I think you have the risk of having these sort of subcultures and promotion within the subculture of whatever that research unit is. (KII 25)

Moreover, research productivity appeared to vary across community sites (spoke) compared to fully affiliated sites because of these subcultures:

The community sites don’t place the same emphasis on research productivity. (ECR FG2)

### Creating a pipeline of skilled and diverse researchers through mentorship, training, and capacity building

Creating a pipeline of skilled and diverse primary care researchers was considered crucial to a high impact research department. Creation of this pipeline depended on leadership commitment to EDIIA in their recruitment and hiring of faculty, mentoring, training, and research capacity building.

Researchers recognized there was a need for greater promotion of equity, diversity, and inclusion by the leadership in the department. Research faculty thought it was important to ensure that the department included researchers who represent the diversity of the population being served. This was important so that the research is relevant to diverse clinical populations.

… but just thinking about who is providing mentorship, to who’s getting, early supports. So, if you’ve got mentorship… And I think that that’s where folks from under-represented groups often don’t have those same kind of… we’d like to see the DFCM on a research program that just reflects the diversity of the province and the country, which we …do not have right now. (MCR FG)

There is an incredible whiteness to the department from the leadership particularly. And that can be very ostracizing for people of colour in the department at multiple different levels, including in research. I’ve been made to feel an outsider by leadership… I don’t think there’s a lot of recognition at the leadership level of what kind of impact that has on the department, and who ends up wanting to and being able to thrive in the department as a researcher. (ECR FG1)

I don’t see a lot of gay people at the… As you go further up in academic medicine, the proportion seems to decrease. And I find that quite striking. And those things are always true for a reason. And, you know, I think we can be intentional about this. (ECR FG1)

Research leaders agreed EDIIA was an important focus for primary care research. Most research leaders indicated that EDIIA as social constructs in research have been integrated into the mission of their department or institution. However, for some this initiative is a ‘work in progress,’ while others have a strong foundation with established guidelines, expectations, training, and support. Several international informants reported that there was a significant number of women in their organization but they had variable success in attracting researchers from minority groups.

I think is a strong foundation of values and commitment to the DEI work in the department. It’s central to our mission. We’ve come up with a guideline… our scholarly work… if you’re including race ethnicity, for example, as a variable of interest, why? So despite this interest, we’ve had a real lack of success in recruiting and also retaining research-oriented faculty of colour. (KII 05)

The equity program… So we have, for example, chronically ill panels and disabled people panel. And we ask them about their opinions about health care issues. So that’s one way to go, for example. And so we have quite regularly an update of the diversity within our institute, or how we can approach it, how we can improve it. We are an organization that has a lot of women. So we don’t have so many people from migrant cultures, for example. So we were thinking about that. Well, we try to be inclusive as we can. And we are all well aware of not only women, but also women with children at home. (KII 10)

You know, how do you have a bit more diversity in the mix? And as a department, we are mainly women. In fact, there’s only kind of handful of men. We’re 75, but we’re mainly women. But in terms of indigeneity, we have tried to do that. But we haven’t… It’s really hard to get the researchers. (KII 17)

Mentorship was identified by all informants as key for creation of a high impact research department. All international research programs had some type of either formal or informal mentorship program for early-career researchers. Mentorship, if well-structured and done consistently with accountable mentors, was perceived to drive research excellence and leave a legacy of people who are better qualified. Research leaders agreed that mentoring was an essential function of their role.

And I think that one of the strengths that came from us was our good track record for mentoring and development, which we’ve been building up… I’m quite strict on making sure that everybody who comes through is aligned [with a mentor]. (KII 02)

Research leaders described advocating for or implementing fellowships for clinicians and providing opportunities for research training through courses or embedding them in existing projects (i.e., learning by doing). Several international leaders had created research capacity in their department through the development of PhD programs in primary care.

Since 2009, we now have academic fellowships which are funded through [Program Name]. So, one fellowship in each medical school in [Country]. So, if you’re a cardiologist, you can go out of the program for two years and do research experience. And that had not been open to general practice. And so, we got it opened up to general practice. (KII 02)

Research leaders recommended that exposure to formal research training should begin in medical school, and that residency be extended to include research training. In addition, they suggested that research training with seed funding, and mentoring and educational programs be embedded to build research capacity.

I think it [research training] has to start out in medical school for primary care, and I think it has to carry all the way through. And the same in the residency program for primary care. It’s extremely difficult in two years to do anything other than their core curriculum. So, I guess there is talk about making it a three year. If that happens, then you might be able to get research involved. (KII 04)

### Building collaborative networks by creating partnerships and building relationships

International informants commented that collaboration was central to the success of their own research department through creation of partnerships and relationships. Collaboration, including international collaboration, was viewed as essential in creating a high impact research department.

We’re embedded in a wider [Name of Institute], and we collaborate more widely. So we’re not insular… We collaborate across the [Name of medical school], and across the university… and we encourage juniors to be very collaborative… (KII 02).

It was really essential to take advantage of the broader institutional resources, build alliances, partnerships, collaborations that would allow us to get started. And so I think that’s always been the continuing dance, is a lot of still collaborations that are outside the department, but then trying to strengthen our internal identity and more internal collaboration. (KII 05)

### Research department supports including funding, and infrastructure and methodological supports

Clinical time buyout was also mentioned as a barrier to balancing research with clinical practice. Several senior scientists commented that they had to cobble together funding from several sources which was stressful and took time away from research. Mid-career researchers were more vulnerable of ‘falling off the cliff’ when funding ran out and/or support was withdrawn, making it difficult for them to return to research.

I think the salary support is a big issue, too. Like just to provide people enough time to be away from clinical work to focus on research in a really substantive way. There are just too few salary supports. And the ones that are there are so competitive that it’s very hard for people to have that. I think the other thing is when people hit a rough patch, like a drought in their funding, that there should be supports to help them through that so that they don’t just kind of fall off the cliff. (MCR FG)

Research faculty found it challenging to obtain protected time for research productivity. From their perspective, a successful research program depends on a department that provide these required supports. For example,

Good programs have focused time to do research. Stretched programs are doing research off the side of their desk… but that generally doesn’t lead to a successful research program. (MCR FG)

To be an established senior researcher, you need to spend a significant amount of time… you need to be a 70 − 80% researcher to be a successful senior researcher to be competitive with other successful senior researchers. Early and mid-level people have little ability to get that protected time because the mechanism…the traditional support has only been 40%. (Senior career researcher (SCR) FG)

They don’t have the financial resources to put towards somebody to protect their time for research. And protecting time is…that’s sort of the history of the research program. It had some great researchers, but the productivity was terrible because they didn’t have protected time to do research. (Key informant interview (KII) 04)

Research leaders agreed that stable funding was essential to a successful research program.

I learned was that we did need to figure out how to finance it. And do it in a way because it can’t be funded as a project-by-project thing because you’ll never create the operations to do that. So you need some funding that is long term. (KII 06)

They don’t have secure funding. So they often don’t commit to it. So we started to create special programs. (KII 17)

International leaders and internal informants confirmed that infrastructure and methodological supports were key enablers of a high impact research program. Research faculty also indicated that skilled infrastructure support was essential so that they could concentrate their efforts on designing research protocols. Most international research programs had several research coordinator positions as well as administrative support.

And so that kind of infrastructure would be really important. And I wonder if there’s any opportunity for methodologic support or research admin support. (KII 19)

A major enabler of research were the extensive skills of research staff. Staff members were key facilitators of research; many had research support skills and expertise, with important networks of relationships that enhanced the programmatic and central areas of human resources and finance.

And a lot of this was engaging staff, not just faculty, but a lot of the research staff to really feel invested, connected. Again, ability to see staff as a shared resource that could move from a project to another across investigator teams based on grant cycles and things like this to really elevate some of the skillset. (KII 05)

I think the key driving factor in upscaling research is definitely relationship building. And even for us as [research administrators], we are interacting with the individuals involved in research to the top level. So, keeping and maintaining that relationship definitely makes an impact of our organization outside the department. (DFCM Staff FG)

Research leaders mentioned that essential methodological and content area expertise were provided by full time non-clinician researchers.

So the vast majority of the people that are the scientists, they are the methodologists. They’re full timers. They clearly have content expertise in their area. But then they have a whole variety of clinician scientists and people that they can work with that are in the field. (KII 06)

However, some research leaders indicated that they were conservative with regards to hiring non-clinician researchers because there were few core-funded opportunities to support these positions. Other research leaders of primary care grew their team recognizing that having PhD researchers was advantageous and ‘ups the game’ of their research program:

We’re very conservative about bringing in non-clinician scientists or PhD scientists just because of the financial implications. (KII 01)

We have recently grown our PhD research investigator team. That has been hugely advantageous. We kind of centred family physicians with a few PhD partners and things that grew up in the programs. We have a wonderful cadre of newer PhD researchers. (KII 05)

### Focused research thematic areas

Research leaders suggested that research programs coalesced around key themes have greater impact. Moreover, research leaders suggested that focusing on ‘big ideas in family medicine’ would help cement a program’s international reputation and would attract philanthropic support. However, they cautioned that research themes that are too narrow may stifle the curiosity that motivates many researchers.

I think you need to think what are your areas of competitive advantage as researchers because of who you have already or because of the institutions you work with … and determine what those are and build those up… that does mean that some people are going to be potentially left out. It’s the only way, to really have a really high impact research program, is to really focus on maybe two or three areas, and look for synergies. (KII 01)

We believe that you can’t be everything to everybody… to be really good at anything and be world renowned. So we have to pick our swim lanes. (KII 06)

Across international informants, there was recognition that context was important in making decisions about thematic research areas. A top-down approach, strategically declaring thematic areas, was especially relevant to institutions reliant on securing donor funding. For departments reliant on ‘soft’ funding, having thematic areas was not as feasible. Instead, efforts were focused on building linkages and identifying opportunities for synergy. For several international departments, thematic areas had developed organically over time arising from shared research interests rather than a ‘top-down’ approach. Relevant thematic areas suggested by external informants included multi-morbidity, continuity of care, chronic disease prevention, and impact of COVID-19. Other suggestions included methodological approaches to research in primary care.

### Macro level: culture and policies

The macro level relates to the overarching culture that shapes the production of high impact research. A key enabler identified was policies related to funding to elevate the importance of primary care research to all major systems and institutions including the national and provincial organizations that fund research, university supports and collaborations, cross-university primary care consortia that govern and shape the health research landscape. Another theme with respect to the overarching culture was the need to re-define the metrics that academic faculties, funders and policy makers use to measure primary care research impact.

### Recognizing the cultural importance of primary care research

Research faculty indicated that the most important factor for the creation of a high impact research department is funding but all agreed that it was a challenge in securing funding for research.

In addition to writing grants, getting rejected…. Challenges are many. Securing funding is really hard. (ECR FG2)

So, it all comes to grants…they start “I’m going to go for an NSERC grant, a CIHR grant^1^” – grants that are uber competitive. They get disappointed when they don’t get one…it turns off a lot of researchers. (MCR FG)

Research leaders noted that primary care research was not a priority for the funding bodies.

Then we need to figure out where we’re going to fund this work. Because this is not something that traditional, research sources fund traditionally. And so we have to figure out where we’re going to get support because our health service delivery partners can’t fund it. CIHR [Canadian Institutes for Health Research] won’t fund it, or has not real priority in it. So where does that funding come from? (KII 06)

However, all informants emphasized the importance of research in primary care. They indicated that as most patient care occurs in primary care and emergency care settings, research on the impact of illness on health systems and on patients cannot be addressed by specialist research but can only be answered by primary care or public health research. The challenge indicated by some informants is that primary care can be perceived as too broad, making it difficult to be heard and attract funding. Informants reiterated that primary care physicians needed to be engaged, involved, informed, at the forefront, and strategically positioned with researchers in other departments. There also needs to be intentional investment in implementation scientists to bring meaningful change to patient care. Some felt that the primary care story needed to be promoted. One such example was “Primary care has the ability to impact health care system needs the best by having continuity of care and relationship-based care.” Such promotion would secure investment in primary care to prevent and manage complex chronic diseases.

The recognition of the importance of primary care research at the macro-, policy-level may contribute to targeting areas of greater need in primary care and improving community health. For example, two high impact research programs have secured funding and built strong collaborative networks with their respective governments that recognize the importance and support primary care research.

We also have in [name of country] something called the [title of] project, where our department started it and managed to get some funding from [name of] government to buy protective time for the 100 most deprived practices in [name of country]…we’ve helped them articulate issues that are important for general practice, especially in areas of socioeconomic deprivation, that have reached policymakers and also have reached practitioners because they’re quite widely disseminated. (KII 02)

So [name of institute] is not a university. It’s an independent institute for primary care- related research. And it started actually by the wish of primary care physicians and informants to have a research institute connected to their interests. And that’s how it was established and how it started. …So we have around 200 employees there. So it’s partly funded by the government, by the Ministry of Health, and partly, it’s based on collaborations with the [name of service] public health services. (KII 10)

### Re-defining the metrics beyond the dimensions of publications, grants, and presentations

Consistent with the researchers’ perspective, research leaders in the project noted that there was a need for different metrics to determine primary care research success. International research leaders commented that their research programs were attempting to find appropriate metrics of high impact research. The Research Excellence Framework developed in the United Kingdom was described by some as a useful approach [35, 36]. All informants commented that research impact should be determined by improvements in the following: health of populations including social determinants of health, health care, health systems, and economic benefits.

And we need different metrics of success… because the research metrics that we typically use in health care don’t work… So it’s looked upon often as sub-standard research. Plus, this is where you have impact. So if you want to be a researcher with impact, you have to work in partnerships… the impact they’ve had in transforming patients…what actually has changed in our system in terms of the way care delivery has happened? (KII 06)

And this is not only about how many publications you have… it’s about potential that you spread. It’s also about… because we have also a societal task… bit more also valuing the societal part. If people write relevant policy reports, it’s also fine. (KII 10)

## Discussion

We found that a high impact research department requires strong support for research from leadership, thematic areas of research to attract donor funding, mentorship, fostering of local, national, and international collaborations, methodological and infrastructure support, and research training and capacity building. These findings are consistent with previous studies that have explored factors associated with successful primary care research programs and research productivity [15, 37–40]. However, as we also found, clinician researchers while highly motivated, driven by curiosity and their commitment to addressing patient care and population health, experienced challenges related to their research productivity including securing funding and protected time for research. Researchers also indicated that their research career pathway was often one of happenstance and a “do-it-yourself” approach.

Researchers in our project believed that they are uniquely positioned in the health care system and are motivated to produce high impact primary care research. They described high impact research in primary care as having real world impact and making a difference, influencing policy, improving population health, and effecting change in health systems. They also indicated a need to move beyond the traditional metrics of measuring research outputs such as grants and publications. This is consistent with evidence of available tools and descriptions that have evolved where alternative metrics attempt to capture, for example, the “changes in the healthy functioning of individuals (physical, psychological, and social aspects of their health), changes to health services, or changes to the broader determinants of health such as reductions in health disparities” [41, 42].

Researchers noted that there was a need for a more supportive research culture at the practice setting where leadership promote the value of primary care research and provide the resources and support needed to produce high impact research. The local research culture was often dependent on the leadership at the practice sites (the spoke) which appeared to have had greater influence on researchers and whether primary care research is supported in comparison to the hub. Our findings identified several shortcomings of organizational structure, the hub and spoke design, and its influence on research productivity which included limited communication and collaboration, and perception of relative paucity of resources at community sites. Organizational culture and leadership support for primary care research appeared to vary across the hub and spoke structure. Findings from other studies suggest that fostering collaboration within and among units, increasing communication, and mentorship planning at the hub has been beneficial and can lead to greater productivity in the hub and spoke model [43, 44].

As noted academic departments play a crucial role in the development of primary care clinician scientist workforce and research production [12–14]. As we found, and supported by other studies, a pipeline of primary care researchers needs to be built through a formalized process to encourage research curiosity, provide access to mentors and rigorous training beginning in medical school and residency [45–48]. In addition, as our findings indicate building a pipeline of primary care researchers addressing EDIIA principles has been challenging but essential because studies have shown that a diverse primary care workforce can improve care experience and population health [49]. In recent years there has been a rapid feminization of the primary care workforce a significant demographic change which is reflected in our findings [50] but greater efforts are needed to create a more diverse workforce of primary care researchers.

All participants viewed research in primary care as critical as most patient care occurs in this setting. This is supported by the literature that primary care is about relationships among physicians, patients, families, and communities; it is the backbone of the health system, and primary care research is needed to inform policy that sustain healthcare delivery and the commitment to improving population health [10, 45, 51–53]. Our project findings, supported by other studies, suggest that it was important to foster an overarching culture that values and supports primary care research through the establishment of strong funding policies by government and funding institutions [46, 52, 54–57]. Improved research funding policies directed to areas of greater need, would provide robust evidence, grounded in a primary care context, suitable for adoption in practice thereby improving patient outcomes in the community [14]. Moreover, to highlight the importance of primary care research and its impact, particularly its social impact which is becoming of increasing significance, we need to move beyond simple bibliometrics as our project participants and prior literature suggests [41, 58, 59].

To facilitate a cultural shift an appropriate environment recognizing the importance of primary care research, supported by re-defined metrics, and factors such as funding and protected time, research training and support, and the opportunity to become research involved needs to be addressed [60]. This cultural shift could influence, with support from academic faculties, funders and policy makers, the structures, functions, and processes required to create a high impact research department in primary care.

## Strengths and limitations

A significant strength of this project includes the framing of the analysis using a socioecological theoretical model [18] that allowed for the examination the primary care research enterprise at three levels of influence including at the macro level not previously explored by other theoretical/conceptual frameworks [37–39]. Another strength was the inclusion of perspectives from a broad range of key informants including several international informants. In addition, the systematic approach employed for data collection and analysis, which included creating and maintaining an audit trail of coded transcripts and memos, the use of a data management software program and the range of expertise represented within the project team strengthened the project process. A limitation of the project was that few faculty members who did not receive department funding participated which would have allowed for a more comprehensive understanding of the researcher experience at the individual level. Another limitation is that the organizational structures of the primary care research programs, as described by the research leaders we interviewed, were diverse and as result, it was difficult to infer from the participant interviews if the structure promoted or hindered the production of high impact research. Finally, due to time limitations, we did not interview anyone from national funding or primary care organizations and therefore the macro level findings were less robust.

## Conclusion

Using a socioecological framework allowed us to identify multiple levels of influence to create a high impact research department in primary care. The interaction of individual and institutional level characteristics including effective leadership influence the creation of high impact research departments in primary care. At the broader macro level, a cultural landscape that stresses the importance of primary care is needed to leverage funding policies for research directed to improving population health. Drawing on our findings, we propose actions at these levels of influence to enhance the importance of primary care research, provide access to resources, research collaborators and mentors, research support infrastructure, training, funding, and staffing.

### Electronic supplementary material

Below is the link to the electronic supplementary material.

**Supplementary Material 1:** Focus group and key informant interview guides

## Data Availability

The datasets used and/or analysed during the current project are available from the corresponding author on reasonable request.

## Abbreviations

DFCM: Department of Family and Community Medicine
UTOPIAN: University of Toronto Practice Based Research Network
TAHSN: Toronto Academic Health Science Network
EDIIA: Equity, diversity, inclusion, indigeneity, and accessibility
FG: Focus Group
KII: Key Informant Interview
EMR: Electronic Medical Record
SCR: Senior Career Researcher
MCR: Mid Career Researcher
ECR: Early Career Researcher

## Footnotes

1 The Canadian Institutes of Health Research (CIHR), the Natural Sciences and Engineering Research Council of Canada (NSERC) and the Social Sciences and Humanities Research Council of Canada (SSHRC), “the Agencies,” are federal government granting agencies entrusted with managing public funds to enable and support the Canadian research enterprise.

## Declarations

### Other: (Received drugs free/discounted for study through open tender process)

Johnson & Johnson, Novartis, Pfizer Inc. MAO was a paid consultant on the Project for part of the time.
