# Supplementary material for: Key informants perspectives on creating a high impact research department in family and community medicine: a qualitative project
Source: BMC Prim Care. 2024 Feb 6;25:52. doi: 10.1186/s12875-024-02288-6 (PMC10845512; doi:10.1186/s12875-024-02288-6)
Supplement: Supplementary file 1 — Supplementary Material 1: Focus group and key informant interview guides [file 12875_2024_2288_MOESM1_ESM.pdf]

Supplemental File 1: Focus Group and Key Informant Interview Guides

Focus Group Guide

<u>Project Title</u>: High Impact Research Programs in Family and Community Medicine: A quality improvement project

Project Lead: Peter Selby MBBS, CCFP(AM), FCFP, dip ABAM.MHSc

<u>Quality Improvement Project Information</u>

You are being invited to participate in a quality improvement project. Your participation is your choice (voluntary). You have the right to choose not to participate, or to stop participating in this project at any time.

The Research Program, under the direction of the Department of Family & Community Medicine (DFCM) Academic Leadership Executive Committee and the Vice Chair Research (Interim) is conducting an environmental scan with the goal of identifying key components of a high impact research department of family and community medicine.

Our Project Questions

1. What are the types of organizational models for a high impact research department in family and community medicine?

2. What factors are associated with high impact research in family and community medicine?

3. What structures, functions, and processes are required to create a high impact research department in family and community medicine?

You are one of approximately 60 faculty members or staff being invited to participate in an electronic focus group to inform this project aimed at strengthening the Research Program of the Department of Family & Community Medicine (DFCM) at the University of Toronto.

High Impact Family Medicine Research Programs: A quality improvement project

Summary of Focus Group Questions

1. How would you describe the role of research in Family Medicine?
2. What motivates you as a researcher?
3. What are the key challenges to becoming a researcher?

4. What are the specific needs or challenges of early career/mid-career/senior scientist?
5. What are the characteristics of a high impact research department?
6. What are your views on key metrics of research impact?
7. The DFCM is committed to instilling an equitable, diverse and inclusive environment as a key part of research capacity development. What would this look like to you?
8. What are your overall impressions of the DFCM Research Program? What advice would you give regarding the future direction of the program?

Key Informant Interview Guide

Project Title: High Impact Research Programs in Family and Community Medicine: A quality improvement project

Project Lead: Peter Selby MBBS, CCFP(AM), FCFP, dip ABAM.MHSc

Quality Improvement Project Information

The Research Program in the Department of Family and Community Medicine at the University of Toronto is conducting an environmental scan to identify key components of a high impact research department. Given your leadership role, we are hoping to gain your insights into the key facilitators of and barriers to a high impact research department.

Your participation is your choice (voluntary). You have the right to choose not to participate, or to stop participating in this project at any time.

Our Project Questions

1. What are the types of organizational models for a high impact research department in family and community medicine?

2. What factors are associated with high impact research in family and community medicine?

3. What structures, functions, and processes are required to create a high impact research department in family and community medicine?

Key informant interview

1. In your view, what are the key factors that have contributed to the success of research at your university/institute?
    a. Probe responses as needed
2. In your leadership role, what do you consider to be the essential functions [with respect to research]?
3. What processes are in place to support junior researchers to reach their potential?
    a. Probe for mentoring programs
4. What do you consider essential in sustaining a culture that supports excellent research?

5. Your Department/Institute is organized under Research Areas of Excellence/Research Groups/Research Themes. How has this organization promoted the creation of high impact research?

6. How does the Department determine the balance between strategic research aligned with themes/areas of excellence and 'investigator-driven' research? Are there incentives for research aligned with strategy?

7. A focus of research in the Department is on health equity. Our research program will focus on equity, diversity and inclusion. What factors have contributed to the Department's success in research focused on health equity?

    a. Probe for researcher hiring and training

    b. Probe for studying vulnerable populations e.g., partnerships

8. What does the Department consider to be the most important indicators of research impact?

9. How does the Department measure and reward success?

    a. How to you make decisions to move resources away from under-performing individuals or teams?

10. With respect to the interface between research and Quality Improvement, how does the Department deal with this interface?

11. Do you have any advice for us as we reassess our research program?
